# What determines value? Exploring value characteristics of novel therapies for digestive system cancers

**DOI:** 10.3389/fimmu.2026.1747256

**Published:** 2026-02-10

**Authors:** Shunlong Ou, Song Wang, Xiaoyi Chen, Huan Li, Chengyang Zhou, Qian Jiang

**Affiliations:** 1 Department of Pharmacy, Sichuan Clinical Research Center for Cancer, Sichuan Cancer Hospital & Institute, Sichuan Cancer Center, University of Electronic Science and Technology of China, Chengdu, China; 2 School of Medicine, University of Electronic Science and Technology of China, Chengdu, China

**Keywords:** ASCO-VF, clinical benefit, digestive system cancers, ESMO-MCBS, factors associated value, novel anti-tumor drugs

## Abstract

**Introduction:**

Despite the growing arsenal of novel anti-tumor drugs for digestive system cancers, concerns persist regarding their true clinical value, as approvals often rely on surrogate endpoints with limited overall survival or quality of life data. This study systematically evaluates the clinical benefit of these drugs in China using standardized value assessment frameworks and identify factors associated with high-value treatments.

**Methods:**

This cross-sectional study analyzed 65 indications from 33 novel anti-tumor drugs included in the Guidelines for the Clinical Application of Novel Anti-tumor Drugs for advanced digestive system cancers. Two researchers independently extracted data from drug approval documents and PubMed literature. Value assessment was performed using ASCO Value Framework v2.0 (advanced disease) and ESMO-Magnitude of Clinical Benefit Scale v2.0 (non-curative). For ASCO-VF, the net health benefit score was calculated based on clinical benefit, toxicity adjustments, and bonus points. ESMO-MCBS grading incorporated clinical benefit magnitude with adjustments for toxicity and quality of life. Statistical analyses included descriptive statistics and association testing with odds ratios for categorical variables.

**Results:**

Among 65 indications, only 12 (18.5%) achieved value thresholds in both frameworks. Immune combinations showed superior value, particularly atezolizumab-bevacizumab in hepatocellular carcinoma (ASCO-VF: 53.4; ESMO-MCBS: grade 5) and toripalimab in esophageal squamous cell carcinoma. Quality of life improvement strongly correlated with value attainment in both frameworks (ASCO-VF OR: 28.00; ESMO-MCBS OR: 27.87). Molecular target detection requirement inversely associated with OS benefit (OR: 0.16). Value heterogeneity was observed across cancer types and treatment lines.

**Conclusions:**

Few novel digestive system cancer drugs demonstrate substantial clinical benefit in both value frameworks. Quality of life documentation is crucial for value assessment. Stakeholders should integrate these value dimensions—survival benefit, toxicity, and quality of life—into clinical and policy decisions to guide treatment selection and resource allocation.

## Introduction

In recent years, digestive system tumors (including colorectal cancer, esophageal squamous cell carcinoma, hepatocellular carcinoma, etc.) have remained highly prevalent worldwide (1). Characterized by complex pathogenesis and poor prognosis, they pose a serious threat to human health. With the rapid development of precision medicine and molecular targeting technologies, novel anti-tumor drugs for digestive system tumors—such as molecular targeted agents and immune checkpoint inhibitors—continue to emerge, providing patients with more personalized treatment options. However, the clinical development and regulatory review and approval of novel drugs often rely on surrogate endpoints such as progression-free survival (PFS) and objective response rate (ORR), while the supporting evidence for critical outcome indicators like overall survival (OS) and quality of life (QoL) remains relatively weak (2–4). International studies indicate that a majority of anticancer drugs approved by the FDA and EMA lack sufficient evidence of OS and QoL improvement (5, 6), a trend that is similarly pronounced in the field of digestive system tumors. Furthermore, to address urgent clinical needs, some drugs are accelerated to market through priority review or conditional approval pathways, resulting in a weak correlation between their clinical value and regulatory standards. This may lead to drugs with uncertain value entering clinical use (7).

On the level of value assessment, the American Society of Clinical Oncology (ASCO) and the European Society for Medical Oncology (ESMO) have developed ASCO Value Framework (ASCO-VF) and the ESMO-Magnitude of Clinical Benefit Scale (ESMO-MCBS), respectively, to systematically quantify drug value across dimensions including survival benefits, quality of life, safety, and economic burden (8, 9). Existing research indicates that clinically meaningful net health benefits have been observed only in a limited proportion of solid tumor drugs (7, 10, 11), while specialized value assessments for novel drugs targeting digestive system tumors remain a gap. The lack of a mechanism for value identification and drug selection in this field may impact the scientific formulation of clinical treatment strategies.

A systematic assessment of the value of novel gastrointestinal cancer drugs is essential. It can identify the most clinically valuable treatments among approved options to inform clinical pathways and drug policies. This study will apply and compare these established multidimensional frameworks to evaluate these drugs, explore factors affecting their value, and support the development of value-driven cancer care strategies. It is important to clarify that this study relies on applying and comparing these existing frameworks rather than proposing a new one.

## Methods

### Data sources

This study focused on the novel anti-tumor drugs included in the Guidelines for the Clinical Application of Novel Anti-tumor Drugs (2024 Edition). Drugs indicated for advanced digestive system tumors were selected, while those for non-digestive system tumors or used in adjuvant therapy were excluded. The literature retrieval process was conducted as follows: First, two researchers systematically retrieved pivotal clinical studies from the marketing approval reports issued by the Center for Drug Evaluation of the National Medical Products Administration, with key studies listed in the drug labeling serving as supplementary sources. Next, trial registration numbers were extracted from the relevant registration documents. Subsequent searches were conducted in PubMed to identify any post-marketing study updates. For agents supported by multiple clinical trials, the study with the largest sample size was selected. Finally, research reports on patient-reported outcomes (PROs) from the associated clinical trials were collected. Any discrepancies were resolved through discussion with a third researcher to reach a consensus.

### Data extraction

For each included trial, two researchers independently extracted data from the literature using a pre-designed structured Excel form. The accuracy and completeness of the extracted information were cross-verified between them. The extracted data included, but were not limited to: drug approval details (accelerated approval or regular approval), reimbursement status, trial characteristics (e.g., trial name, tumor type, study design, phase, treatment regimen, and line of therapy), primary efficacy endpoints (OS, PFS and their HR values for randomized controlled trials; PFS, ORR and duration of response for single-arm studies), as well as information on toxicity and QoL.

OS benefit was defined as a statistically significant difference observed between the trial and control groups (4). QoL benefit was defined as a significant advantage in PROs for the intervention group compared to the control group in randomized controlled trials, or a significant improvement from baseline after treatment in single-arm studies (4).

### Value scoring

We employed the ASCO-VF version 2.0 (advanced disease) and ESMO-MCBS version 2.0 (non-curative) to quantify the value scores of the included indications (8, 9). The ASCO-VF is applicable only to phase II or III randomized controlled trial (RCT) and consists of three modules: clinical benefit, toxicity, and bonus points. These are ultimately combined into a net health benefit (NHB) score, which is a continuous measure ranging from -20 to 180. While the framework itself does not specify a definitive value threshold, this study referenced our previous research and defined an NHB score ≥ 38.2 as meeting the value threshold (11). The clinical benefit score is calculated as (1 − Hazard Ratio (HR)) × 100 × weight (with weights of 1 for OS, 0.8 for PFS) and (Complete Response rate + Partial Response rate) × 100 × 0.7 for ORR. When OS data are immature, PFS is used, and then ORR. The toxicity score is derived from the percentage difference in total toxicity points between the intervention and control groups, multiplied by 20. The NHB score is adjusted by subtracting the toxicity score for a more toxic intervention or adding it for a more toxic control. Bonus points comprise: long-term survival (up to 20 points; OS weight 1, PFS 0.8), cancer-related symptom improvement (10 points), QoL gain (10 points), and the percentage improvement in treatment-free interval × 20.

The ESMO-MCBS designed for positive clinical trials, integrates evaluations from two modules: clinical benefit and toxicity/QoL, to produce a final grade on a 1–5 scale. Grades may be elevated by one level upon documented improvement in specific grade 3–4 adverse events or QoL; conversely, an improvement confined to PFS that fails to translate into OS benefit or QoL enhancement may result in a one-level downgrade. The clinical benefit grade is assigned based on where the lower limit of the HR’s 95% confidence interval falls within pre-defined thresholds. For example, with a control median PFS <6 months and HR ≤0.65, a PFS gain of ≥1.5 months corresponds to grade 3; a smaller gain results in grade 2. Grades 4 and 5 are explicitly considered to meet the value threshold for therapies in advanced cancer.

Since this study considered only non-curative treatments, we employed the ASCO-VF for advanced disease and utilized ESMO-MCBS forms 2a, 2b, 2c, and 3 for scoring.

### Statistical analysis

Descriptive statistics were employed to summarize the data, with categorical variables presented as proportions and continuous variables described using median and range. We investigated the associations between trial characteristics and OS, QoL, as well as the threshold for value benefit. Statistical significance for these associations was initially assessed using either the Chi-square test or Fisher’s exact test, as appropriate. For 2×2 contingency tables, odds ratios (OR) were subsequently calculated to quantify the strength of the associations. The results are presented as odds ratios along with their corresponding 95% confidence intervals. All analyses were performed using R language (version 4.3.1). All hypothesis tests were two-sided, with the significance level set at α = 0.05.

## Results

### Characteristics of included trials

In this analysis, 33 novel anti-tumor drugs covering 65 indications were included (12–87). Of these indications, 83.1% were supported by Phase III RCT, providing compelling evidence, and nearly 70% of the drugs were monoclonal antibodies or other large-molecule agents. In terms of regulatory approval and market access, 43.1% of the indications were approved through accelerated pathways, while 61.5% were included in the National Reimbursement Drug List (NRDL)—comprising 21.5% formally listed and 40.0% granted temporary NRDL access via national price−negotiated. Additionally, over 70% of indications required no molecular target detection, and more than half were approved for first-line treatment, suggesting notable clinical convenience and potential for earlier use in treatment algorithms. **Table 1**; **Supplementary Table S1** present the detailed characteristics of the included trials.

**Table 1:** Characteristics of included indications and trails.

Characteristics	n (%)
Drug categories	
Protein kinase inhibitors	20 (30.8)
Monoclonal antibodies and antibody-drug conjugates	45 (69.2)
Originator drug	
Domestic	31 (47.7)
Imported	34 (52.3)
Indications	
Colorectal cancer	10 (15.4)
Esophageal squamous cell carcinoma	10 (15.4)
Esophageal or gastric or gastroesophageal junction cancer	14 (21.5)
Hepatocellular carcinoma	16 (24.6)
Gastrointestinal stromal tumor	5 (7.7)
Others	10 (15.4)
Molecular target detection	
Yes	18 (27.7)
No	47 (72.3)
Reimbursement status	
Listed on the NRDL	14 (21.5)
National price-negotiated	26 (40.0)
Out-of-pocket	25 (38.5)
Approval types	
Accelerated	28 (43.1)
Regular	37 (56.9)
Line of treatment	
First line	34 (52.3)
Later lines	31 (47.7)
Study phases	
I–II	12 (18.5)
III	53 (81.5)
Study design	
Randomized controlled trials	54 (83.1)
Single-arm trials	11 (16.9)
Blinding	
Yes	34 (52.3)
No	31 (47.7)

NRDL, National Reimbursement Drug List.

Among the 54 RCTs evaluated using the ACSO-VF framework, the NHB scores ranged from 1.0 to 74.0, with a median of 32.5 (IQR 17.5-41.0). Of these, 18 (33.3%) treatments met the value threshold (**Table 2**). When the entire cohort of 54 RCTs and 11 single-arm studies was assessed with the ESMO-MCBS, 26 (40.0%) treatments met the value threshold.

**Table 2:** Clinical benefit of novel anti-tumor drugs for digestive system cancers.

NO.	Drug	Indication	Intervention	Control group	Line of treatment	ASCO-NHB	ESMO-MCBS
1	Pembrolizumab (12, 13)	Colorectal cancer	Pembrolizumab	CT	1	61.7	4
2	Bevacizumab (14)	Colorectal cancer	Bevacizumab+CT	CT	1	34	3
3	Cetuximab (15)	Colorectal cancer	Cetuximab+CT	CT	1	15.9	2
4	Cetuximab (16)	Colorectal cancer	Cetuximab+CT	cetuximab	≥2	6.6	1
5	Envafolimab (17)	Colorectal cancer	Envafolimab	/	≥2	/	3
6	Serplulimab (18)	Colorectal cancer	Serplulimab	/	≥2	/	4
7	Pucotenlimab (19)	Colorectal cancer	Pucotenlimab	/	≥2	/	3
8	Fruquintinib (20)	Colorectal cancer	Fruquintinib	placebo	3	15	3
9	Regorafenib (21)	Colorectal cancer	Regorafenib	placebo	3	25	3
10	Tislelizumab (22)	Colorectal cancer	Tislelizumab	/	≥3	/	3
11	Sintilimab (23)	Esophageal squamous cell carcinoma	Sintilimab+CT	CT	1	35.2	3
12	Camrelizumab (24)	Esophageal squamous cell carcinoma	Camrelizumab+CT	CT	1	38.1	3
13	Tislelizumab (25)	Esophageal squamous cell carcinoma	Tislelizumab+CT	CT	1	32.7	4
14	Toripalimab (26)	Esophageal squamous cell carcinoma	Toripalimab+CT	CT	1	39.1	4
15	Nivolumab (27)	Esophageal squamous cell carcinoma	Nivolumab+CT	CT	1	39.9	3
16	Sugemalimab (28)	Esophageal squamous cell carcinoma	Sugemalimab+CT	CT	1	24.5	4
17	Serplulimab (29)	Esophageal squamous cell carcinoma	Serplulimab+CT	CT	1	28.3	4
18	Camrelizumab (30)	Esophageal squamous cell carcinoma	Camrelizumab	CT	2	73.2	4
19	Tislelizumab (31, 32)	Esophageal squamous cell carcinoma	Tislelizumab	CT	2	70	4
20	Pembrolizumab (33, 34)	Esophageal squamous cell carcinoma	Pembrolizumab	CT	2	45.9	4
21	Pembrolizumab (35, 36)	Esophageal or Gastroesophageal Junction Cancer	Pembrolizumab+CT	CT	1	32.3	3
22	Sintilimab (37)	Gastric or gastroesophageal junction cancer	Sintilimab+CT	CT	1	43	2
23	Tislelizumab (38)	Gastric or gastroesophageal junction cancer	Tislelizumab+CT	CT	1	18.9	2
24	Trastuzumab (39, 40)	Gastric or gastroesophageal junction cancer	Trastuzumab+CT	CT	1	43.5	4
25	Nivolumab (41, 42)	Gastric or gastroesophageal junction cancer	Nivolumab+CT	CT	1	39.1	2
26	Sugemalimab (43)	Gastric or gastroesophageal junction cancer	Sugemalimab+CT	CT	1	26.0	3
27	Cadonilimab (44)	Gastric or gastroesophageal junction cancer	Cadonilimab+CT	CT	1	31.3	4
28	Pembrolizumab (45)	Gastric or gastroesophageal junction cancer	Pembrolizumab+trastuzumab+CT	trastuzumab+CT	1	11.7	2
29	Pembrolizumab (46)	Gastric or gastroesophageal junction cancer	Pembrolizumab+CT	CT	1	16.1	1
30	Ramucirumab (47, 48)	Gastric or gastroesophageal junction cancer	Ramucirumab+CT	CT	2	18.7	2
31	Apatinib (49)	Gastric or gastroesophageal junction cancer	Apatinib	placebo	≥3	22.8	2
32	Nivolumab (50)	Gastric or gastroesophageal junction cancer	Nivolumab	placebo	≥3	37	1
33	Vedituximab (51)	Gastric or gastroesophageal junction cancer	Vedituximab	/	≥3	/	2
34	Trastuzumab deruxtecan (52)	Gastric or gastroesophageal junction cancer	Trastuzumab deruxtecan	CT	≥3	34.9	4
35	Sorafenib (53)	Hepatocellular carcinoma	Sorafenib	placebo	1	12	3
36	Lenvatinib (54, 55)	Hepatocellular carcinoma	Lenvatinib	sorafenib	1	15.9	3
37	Donafenib (56)	Hepatocellular carcinoma	Donafenib	sorafenib	1	17.6	2
38	Atezolizumab (57, 58)	Hepatocellular carcinoma	Atezolizumab+bevacizumab	sorafenib	1	53.4	5
39	Sintilimab (59)	Hepatocellular carcinoma	Sintilimab+bevacizumab	sorafenib	1	41	4
40	Camrelizumab (60)	Hepatocellular carcinoma	Camrelizumab+apatinib	sorafenib	1	43.3	4
41	Tislelizumab (61, 62)	Hepatocellular carcinoma	Tislelizumab	sorafenib	1	35	4
42	Apatinib (60)	Hepatocellular carcinoma	Apatinib+camrelizumab	sorafenib	1	43.3	4
43	Bevacizumab (59)	Hepatocellular carcinoma	Bevacizumab+sintilimab	sorafenib	1	41	4
44	Bevacizumab (57, 58)	Hepatocellular carcinoma	Bevacizumab+atezolizumab	sorafenib	1	53.4	5
45	Regorafenib (63)	Hepatocellular carcinoma	Regorafenib	placebo	2	20.8	3
46	Camrelizumab (64)	Hepatocellular carcinoma	Camrelizumab	/	2	/	1
47	Tislelizumab (65)	Hepatocellular carcinoma	Tislelizumab	/	2	/	1
48	Pembrolizumab (66)	Hepatocellular carcinoma	Pembrolizumab	placebo	2	1	2
49	Apatinib (67)	Hepatocellular carcinoma	Apatinib	placebo	≥2	5.5	2
50	Ramucirumab (68)	Hepatocellular carcinoma	Ramucirumab	placebo	2	9	1
51	Imatinib (69)	Gastrointestinal stromal tumor	Imatinib	/	1	/	4
52	Avapritinib (70)	Gastrointestinal stromal tumor	Avapritinib	/	1	/	3
53	Sunitinib (71)	Gastrointestinal stromal tumor	Sunitinib	placebo	2	31.5	4
54	Regorafenib (72, 73)	Gastrointestinal stromal tumor	Regorafenib	placebo	3	3	2
55	Ripretinib (74, 75)	Gastrointestinal stromal tumor	Ripretinib	placebo	4	74	5
56	Pembrolizumab (76)	Biliary tract cancer	Pembrolizumab+CT	CT	1	17.2	1
57	Durvalumab (77, 78)	Biliary tract cancer	Durvalumab+CT	CT	1	23.7	2
58	Pemigatinib (79)	Biliary tract cancer	Pemigatinib	/	≥2	/	3
59	Surufatinib (80)	Extrapancreatic neuroendocrine tumor	Surufatinib	placebo	≥2	34.6	4
60	Serplulimab (18)	Gastric cancer	Serplulimab	/	3	/	4
61	Everolimus (81, 82)	Non-functional neuroendocrine tumor of the lung or gastrointestinal tract	Everolimus	placebo	2	16	2
62	Everolimus (83)	Pancreatic neuroendocrine tumor	Everolimus	placebo	1	38.3	4
63	Sunitinib (84, 85)	Pancreatic neuroendocrine tumor	Sunitinib	placebo	2	20.3	3
64	Nimotuzumab (86)	Pancreatic Cancer	Nimotuzumab+CT	CT	2	34	1
65	Surufatinib (87)	Pancreatic neuroendocrine tumor	Surufatinib	placebo	≥2	54.1	4

CT, chemotherapy; /: Not applicable.

### ASCO-VF and ESMO-MCBS scores for colorectal cancer

First-line pembrolizumab was the only regimen that achieved high value in both tools, with an ASCO-VF NHB score of 61.7 and an ESMO-MCBS grade of 4 (**Table 2**; **Supplementary Tables S2, S3**). Other first-line biologics, including bevacizumab and cetuximab (both combined with chemotherapy), yielded ASCO-VF scores (34.0 and 15.9, respectively) and ESMO-MCBS grade 3, falling short of the value threshold in both frameworks.

Among later-line therapies, third-line regorafenib and fruquintinib showed meaningful survival benefits (OS HR: 0.55 and 0.65) and attained ESMO-MCBS grade 3, but their ASCO-VF scores failed to meet the value threshold (25 and 15) due to toxicity penalties and lack of bonus points. Serplulimab, a domestically developed programmed cell death-1 (PD-1) inhibitor, reached ESMO-MCBS grade 4 in later-line settings, whereas other similar agents, including tislelizumab, envafolimab, and pucotenlimab, received ESMO-MCBS grade 3.

### ASCO-VF and ESMO-MCBS scores for esophageal squamous cell carcinoma

Among first-line regimens combining PD-1 inhibitors with chemotherapy, toripalimab plus chemotherapy demonstrated the highest clinical benefit under both frameworks, achieving an ESMO-MCBS grade 4 and an ASCO-VF NHB score of 39.1. Tislelizumab, sugemalimab, serplulimab plus chemotherapy also attained ESMO-MCBS grade 4 but but failed to meet the value threshold on the ASCO-VF framework, with scores ranging from 24.5 to 32.7.

In later-line settings, PD-1 monotherapy demonstrated enhanced value. Both camrelizumab and tislelizumab in second-line treatment achieved high ASCO-VF scores (73.2 and 70.0 respectively) along with ESMO-MCBS grade 4, representing meaningful clinical benefit under both frameworks. Pembrolizumab in CPS≥10 patients also attained ESMO-MCBS grade 4 with an ASCO-VF score of 45.9.

### ASCO-VF and ESMO-MCBS scores for gastroesophageal junction cancer

Among first-line treatments, trastuzumab combined with chemotherapy achieved an ESMO-MCBS grade 4 and an ASCO-VF NHB score of 43.5, representing one of the few regimens to meet value thresholds in both frameworks. Similarly, cadonilimab plus chemotherapy attained ESMO-MCBS grade 4 with an ASCO-VF score of 31.3. Sintilimab plus chemotherapy showed a moderate ESMO-MCBS grade 2 but reached the ASCO value threshold with a score of 43, largely due to the improvement in long-term survival rates.

In later-line settings, trastuzumab deruxtecan demonstrated substantial clinical benefit with an ESMO-MCBS grade 4, though its ASCO-VF score of 34.9 fell slightly below the meaningful benefit threshold. Nivolumab monotherapy in third-line treatment achieved an ASCO-VF score of 37, approaching the value threshold, despite a low ESMO-MCBS grade 1.

### ASCO-VF and ESMO-MCBS scores for hepatocellular carcinoma

In the value assessment of hepatocellular carcinoma (HCC) therapies, immune checkpoint inhibitor-based combinations demonstrated superior value compared to tyrosine kinase inhibitor monotherapies across both ASCO-VF and ESMO-MCBS.

Among first-line regimens, atezolizumab plus bevacizumab achieved the highest value recognition with an ESMO-MCBS grade 5 and an ASCO-VF NHB score of 53.4, representing the most substantial clinical benefit. Similarly, bevacizumab plus sintilimab and camrelizumab plus apatinib regimens attained ESMO-MCBS grade 4 with ASCO-VF scores of 41 and 43.3 respectively, all meeting meaningful benefit thresholds in both evaluation systems.

Traditional TKIs showed limited value across both frameworks. Sorafenib, regorafenib, and lenvatinib achieved only ESMO-MCBS grade 3 with ASCO-VF scores (12-20.8), failing to reach meaningful benefit thresholds. Later-line monotherapies including pembrolizumab, apatinib, and ramucirumab demonstrated particularly poor performance in ASCO-VF assessments (scores 1-9) due to significant toxicity penalties.

### ASCO-VF and ESMO-MCBS scores for gastrointestinal stromal tumours

In the value assessment of gastrointestinal stromal tumors (GIST), ripretinib emerged as the only regimen demonstrating substantial clinical benefit under both evaluation frameworks, achieving an ESMO-MCBS grade 5 and an ASCO-VF NHB score of 74.0 in the fourth-line setting. This represents the highest value assessment among all GIST therapies evaluated.

Among earlier-line treatments, imatinib attained an ESMO-MCBS grade 4 in the first-line setting based on durable response, though it was not assessed using the ASCO-VF framework. Avapritinib in the first-line setting achieved only ESMO-MCBS grade 3, below the value threshold.

Later-line therapies demonstrated variable performance. Regorafenib failed to reach meaningful benefit thresholds in either framework, with an ESMO-MCBS grade 2 and ASCO-VF score of 3. Sunitinib in the second-line showed discordant results, achieving ESMO-MCBS grade 4 but falling short of the ASCO-VF threshold with a score of 31.5.

### Associations with OS, QoL, and clinical benefit

**Table 3** shows the associations with OS, QoL, ASCO-VF, ESMO-MCBS benefit. For OS benefit, a statistically significant inverse association was observed with molecular target detection requirement (OR 0.16, 95% CI 0.03-0.83, P = 0.04), indicating that therapies requiring companion diagnostics were less likely to demonstrate significant OS improvements. No other characteristics showed significant associations with OS benefit. No significant associations were found between any of the examined characteristics and QoL benefit.

**Table 3:** Associations With OS, QoL and clinical benefit.

Variable	OS benefit		QoL benefit		ASCO-VF benefit		ESMO-MCBS benefit	
	OR (95% CI)	*P* value	OR (95% CI)	*P* value	OR (95% CI)	*P* value	OR (95% CI)	*P* value
Monoclonal antibodies and antibody-drug conjugates (vs Protein kinase inhibitors)	3.30 (0.70–15.64)	0.19	2.57 (0.51–13.01)	0.32	1.98 (0.54–7.28)	0.36	1.36 (0.46–4.05)	0.58
Imported (vs Domestic)	0.16 (0.02–1.45)	0.12	1.35 (0.38–4.79)	0.89	0.89 (0.29–2.80)	0.85	0.51 (0.19–1.40)	0.19
Molecular target detection (vs not)	0.16 (0.03–0.83)	0.04	1.39 (0.36–5.36)	0.72	0.83 (0.19–3.67)	1.00	0.94 (0.31–2.85)	0.91
Reimbursement Status (vs not)	0.46 (0.08–2.56)	0.45	0.56 (0.16–1.98)	0.36	1.70 (0.54–5.38)	0.37	1.00 (0.36–2.77)	1.00
First line (vs later line)	3.11 (0.65–14.85)	0.24	1.35 (0.38–4.79)	0.64	2.33 (0.69–7.89)	0.17	1.87 (0.68–5.13)	0.22
Regular approval (vs accelerated approval)	2.07 (0.45–9.53)	0.43	0.47 (0.13–1.67)	0.24	0.48 (0.15–1.57)	0.22	0.81 (0.30–2.21)	0.68
RCT (vs single-arm)	/		0.53 (0.12–2.41)	0.41	/		1.98 (0.47–8.29)	0.50
Phase III (vs Phase I–II )	/		0.53 (0.12–2.41)	0.41	/		1.42 (0.38–5.31)	0.75
Blinding (vs not)	0.92 (0.19–4.35)	1.00	0.61 (0.14–2.72)	0.68	0.09 (0.03–0.35)	<0.01	0.39 (0.14–1.08)	0.07
OS Benefit	/		1.60 (0.17–14.90)	1.00	3.75 (0.42–33.41)	0.41	5.54 (0.63–49.02)	0.13
QoL Benefit	1.60 (0.17–14.90)	1.00	/		28.00 (3.12–251.30)	<0.01	27.87(3.30–235.10)	<0.01

OS, Overallsurvival; QoL, Quality of life; OR, Odds ratios; CI: /: Not applicable.

For clinical benefit as measured by the ASCO-VF framework, double-blind trial design showed a strong negative association (OR 0.09, 95% CI 0.03-0.35, P<0.01), while QoL benefit demonstrated a strong positive association (OR 28.00, 95% CI 3.12-251.30, P<0.01). Similarly, for the ESMO-MCBS framework, QoL benefit showed a substantial positive association with substantial clinical benefit (OR 27.87, 95% CI 3.30-235.10, P<0.01). No other factors were significantly associated with ESMO-MCBS benefit.

These findings identify distinct patterns of association across value dimensions, with QoL improvement emerging as a critical factor for substantial clinical benefit in both assessment frameworks, while molecular target detection requirement and blinded trial design showed inverse associations with OS benefit and ASCO-VF benefit, respectively.

## Discussion

This study conducted a systematic value assessment of 65 indications associated with 33 novel anti-tumor drugs, revealing both consistencies and discrepancies in value measurement for digestive system tumors between the ASCO-VF and ESMO-MCBS. The results demonstrate that only a minority of treatment regimens simultaneously met the “meaningful clinical benefit” threshold in both frameworks, highlighting the complexity of contemporary oncology drug value assessment and its dependence on the chosen evaluation methodology (88, 89).

### Complementarity and clinical application of value assessment frameworks

The distinct focuses of the two frameworks provide complementary references for clinical decision-making at different levels. This divergence stems from their inherent design purposes: the ESMO-MCBS is designed as a population-level tool to grade the objective magnitude of clinical benefit and strength of evidence, primarily to inform public health policy; whereas the ASCO-VF NHB score is intended as an individual-level aid to guide shared doctor-patient decision-making by incorporating a broader set of patient-centered value dimensions, including toxicity, QoL, and treatment burden. Consequently, while both frameworks can correlate with outcomes like QoL—as observed in our analysis—their primary emphasis differs. The ESMO-MCBS prioritizes quantifiable survival gains and evidence hierarchy within its scoring, while the ASCO-VF explicitly integrates and weighs a wider spectrum of factors relevant to individual patient choices (8, 9, 90). This difference is particularly evident in the assessment of Esophageal Squamous Cell Carcinoma (ESCC): camrelizumab and tislelizumab in the second-line setting achieved high ASCO-VF scores (73.2 and 70.0, respectively) (30–32), primarily due to bonuses for quality of life and palliation, while also receiving ESMO-MCBS grade 4. Conversely, in colorectal cancer, although fruquintinib and regorafenib demonstrated significant survival benefits (HR 0.65 and 0.55), their ASCO-VF scores were low due to toxicity penalties (20, 21). Therefore, the lack of perfect concordance between the frameworks is not a limitation but rather a reflection of their complementary roles. For China, a synergistic application is warranted: the ESMO-MCBS can provide a high-level, evidence-based benchmark for drug evaluation and NRDL negotiations, while the ASCO-VF can offer a structured approach for clinicians and patients to weigh benefits against risks and burdens in individual cases, especially when multiple therapeutic options exist. Given their differing assessment orientations, clinicians should individualize the choice of framework based on treatment goals—such as survival prolongation versus toxicity reduction—to support personalized decision-making (91).

### Identification of high-value treatment modalities across cancer types

This study successfully identified treatment regimens with outstanding value across different cancer types. In HCC, atezolizumab plus bevacizumab demonstrated exceptional performance, achieving both ESMO-MCBS grade 5 and an ASCO-VF score of 53.4, establishing its benchmark status in first-line treatment for advanced HCC (57, 58). In GIST, ripretinib set a cross-cancer type value assessment record in the fourth-line setting (ESMO-MCBS grade 5, ASCO-VF score 74), reflecting the breakthrough progress in late-line drug innovation (74, 75). Toripalimab plus chemotherapy in ESCC and trastuzumab plus chemotherapy in gastroesophageal junction cancer both reached the value threshold in both frameworks (26, 39, 40); these regimens share the common characteristic of balancing efficacy, safety, and quality of life.

### Analysis of the relationship between endpoints and value assessment

This study systematically analyzed the association between different endpoints and value assessment outcomes. Notably, improvements in QoL showed strong correlations with achieving value thresholds in both frameworks (ASCO-VF OR 28.00, ESMO-MCBS OR 27.87), underscoring the central role of PROs in value assessment (11, 92). However, it is important to highlight that 25 (38.5%) trials in this study did not report QoL data, reflecting that the measurement of PROs has not yet received sufficient attention in current oncology drug development. Another counterintuitive finding was that drugs requiring molecular target detection showed a negative correlation with OS benefit (OR 0.16), potentially indicating heterogeneity in biomarker-selected populations or limitations of companion diagnostics (93). In our assessment, drugs receiving accelerated approval (typically based on surrogate endpoints such as PFS and ORR) showed no correlation with value benefit, indicating a disconnect between initial surrogate endpoint gains and ultimate value attainment. Drugs approved based on PFS often failed to demonstrate significant OS benefit, suggesting that surrogate endpoints should be treated cautiously in value assessment (5, 94). Of note, we found that open-label studies were associated with ASCO-VF benefit, raising concerns about determination bias (95).

### Evolution of treatment line and value patterns

The study found the impact of treatment line on value patterns. In esophageal cancer, the value scores of PD-1 inhibitor monotherapy in the second-line setting were significantly higher than those of first-line combination regimens, mainly due to the accumulation of quality of life and palliative care bonus points in later lines. In contrast, first-line treatments generally demonstrated more stable value performance in colorectal and gastric cancers. These line-specific differences offer practical insights for clinical decision-making, suggesting that treatment choices should be tailored to therapeutic priorities—such as rapid tumor control in the first line versus quality-of-life preservation in later lines. Furthermore, they highlight the need for line-specific value assessment in drug reimbursement and resource allocation, as a therapy may demonstrate high value in later-line settings even if its first-line performance is modest. Finally, the strong association between quality of life and value in later lines underscores the importance of incorporating PRO as key endpoints in trials of later-line therapies. This line-aware perspective provides an important reference for evaluating value in line-specific treatment strategies.

### Value implications of approval pathways and insurance coverage

The accelerated approval pathway offers the promise of timely access to innovative therapies, particularly for advanced-stage patients lacking effective treatment options. However, this benefit comes with inherent uncertainties. For instance, while immune-combination regimens in HCC showed excellent value (57, 58), drugs approved based on single-arm trials may prove to have limited efficacy in subsequent studies, potentially exposing patients to uncertainties in therapeutic outcomes and toxicity risks (92). Therefore, to safeguard public health, clinicians must carefully evaluate the quality of evidence, and patients should make treatment decisions on a fully informed basis, thereby maximizing the benefits of the accelerated approval pathway while minimizing its potential risks.

Analysis of insurance coverage revealed that not all drugs meeting the value threshold received insurance coverage, while some drugs not meeting the value threshold obtained temporary insurance access, reflecting the incomplete consistency between value assessment and reimbursement policies.

### Practical implications and future directions for value assessment

The application of value frameworks in practice requires contextual interpretation. ASCO-VF and ESMO-MCBS serve complementary roles—the former aids individualized decision-making by integrating toxicity and quality of life, whereas the latter supports population-level policy with its focus on survival benefit and evidence strength (90). Discordant results between frameworks reflect different value dimensions and should prompt stakeholders to explicitly weigh priorities (e.g., survival vs.QoL). Current frameworks do not fully capture important real−world elements such as treatment convenience, financial toxicity, or caregiver burden (96). Future assessments should systematically integrate patient−reported outcomes and broaden scope to include these factors. Ultimately, these frameworks serve as essential guides for structured deliberation but must be complemented by multidisciplinary judgment to determine true value in specific clinical and health system contexts.

## Limitations

This study has several limitations. First, the value assessment frameworks derive scores based on the specific comparator used in pivotal trials. This poses a contextual challenge when the comparator is a high-cost drug not reimbursed in a given setting, as the measured benefit may not accurately reflect the new drug’s incremental value over the locally accessible and affordable standard of care, potentially affecting the interpretation of its value for domestic policy. Second, our analysis relies on surrogate endpoints for some assessments, faces the inherent evidence constraints of single-arm trials, and lacks long-term follow-up data for a number of included studies. Additionally, missing QoL data in a significant proportion of trials may have led to an underestimation of true clinical value within frameworks that prioritize patient-centered outcomes.

## Conclusion

Value assessment based on ASCO-VF and ESMO-MCBS provides multi-dimensional evidence support for the clinical positioning of gastrointestinal cancer drugs. Immune-combination therapies have established treatment standards in several cancer types. QoL improvement serves as a key bridge connecting the two value assessment systems and should be given full attention in future drug development and value assessment. It is recommended that clinical decision-makers, payers, and regulatory agencies adopt multi-dimensional value frameworks in drug assessment, balancing multiple value dimensions such as survival benefit, toxicity, and QoL, ultimately maximizing patient benefit.

## Data Availability

The original contributions presented in the study are included in the article/**Supplementary Material**. Further inquiries can be directed to the corresponding author.
